# Electrophysiological Heterogeneity of Fast-Spiking Interneurons: Chandelier versus Basket Cells

**DOI:** 10.1371/journal.pone.0070553

**Published:** 2013-08-12

**Authors:** Nadezhda V. Povysheva, Aleksey V. Zaitsev, Guillermo Gonzalez-Burgos, David A. Lewis

**Affiliations:** 1 Department of Psychiatry, University of Pittsburgh, Pittsburgh, Pennsylvania, United States of America; 2 Department of Neuroscience, University of Pittsburgh, Pittsburgh, Pennsylvania, United States of America; 3 Institute of Evolutionary Physiology and Biochemistry of the Russian Academy of Sciences, Saint-Petersburg, Russia; Northwestern University, United States of America

## Abstract

In the prefrontal cortex, parvalbumin-positive inhibitory neurons play a prominent role in the neural circuitry that subserves working memory, and alterations in these neurons contribute to the pathophysiology of schizophrenia. Two morphologically distinct classes of parvalbumin neurons that target the perisomatic region of pyramidal neurons, chandelier cells (ChCs) and basket cells (BCs), are generally thought to have the same “fast-spiking” phenotype, which is characterized by a short action potential and high frequency firing without adaptation. However, findings from studies in different species suggest that certain electrophysiological membrane properties might differ between these two cell classes. In this study, we assessed the physiological heterogeneity of fast-spiking interneurons as a function of two factors: species (macaque monkey vs. rat) and morphology (chandelier vs. basket). We showed previously that electrophysiological membrane properties of BCs differ between these two species. Here, for the first time, we report differences in ChCs membrane properties between monkey and rat. We also found that a number of membrane properties differentiate ChCs from BCs. Some of these differences were species-independent (e.g., fast and medium afterhyperpolarization, firing frequency, and depolarizing sag), whereas the differences in the first spike latency between ChCs and BCs were species-specific. Our findings indicate that different combinations of electrophysiological membrane properties distinguish ChCs from BCs in rodents and primates. Such electrophysiological differences between ChCs and BCs likely contribute to their distinctive roles in cortical circuitry in each species.

## Introduction

Several, often alternative, approaches have been used to classify cortical inhibitory neurons, or interneurons. These approaches have generally emphasized qualitative differences in features such as morphology, intrinsic physiological properties, neurochemical content, or sources and targets of synaptic inputs and outputs, respectively [1]. Measures of intrinsic physiological properties have discriminated a fast-spiking (FS) type of interneuron based on an unmistakably short action potential, or “fast spike” [2]–[4]. These neurons also have the characteristic properties of high frequency firing without adaptation in firing rate, short membrane time constant and large amplitude of hyperpolarization following action potential firing (“afterhyperpolarization”). The majority of FS interneurons express the Ca^2+^-binding protein parvalbumin (PV) [5]–[7].

Yet, FS interneurons do not constitute a homogeneous group, and include two morphologically distinct cell types: basket cells (BCs) and chandelier cells (ChCs). These morphological differences are truly striking and easily recognizable. BCs have large axonal arbors that spread predominantly parallel to the pial surface, whereas ChCs have a more variable spread of axons which furnish vertically organized boutons that form axonal cartridges. Both cell types target the perisomatic region of pyramidal cells, although BCs innervate the soma and proximal dendrites whereas ChCs innervate the axon initial segment [8].

In addition, recent reports have highlighted important functional differences between BCs and ChCs. In contrast to the BCs which consistently provide inhibitory hyperpolarizing outputs to pyramidal cells, GABA neurotransmission from ChCs may have an excitatory depolarizing effect on pyramidal cells in quiescent circuits [9]–[11], but see [12], [13]. ChCs and BCs also differ in the sources of their excitatory inputs; in layer 2/3 of the rodent neocortex, FS BCs receive strong excitatory inputs from layer 2/3 and 4, whereas ChCs receive strong excitatory inputs from layers 2/3 and 5A [14].

In addition to these differences in their functional roles, BCs and ChCs have been reported to exhibit different membrane properties in studies involving mice [10], ferrets [15], or monkeys [16]–[18]. However, the reported differences are inconsistent and seem to vary across species. In order to clarify these issues, here, we compared the intrinsic membrane properties of ChCs and BCs in the rat and macaque monkey prefrontal cortex (PFC), the cortical region that differs most substantially between these two species [19]. In our previous studies, we examined the electrophysiological classification of interneurons in monkey PFC [6], [16]–[18], and compared BCs in rat and monkey PFC [20]. Here, for the first time, we compared properties of ChCs from monkey and rat and report interspecies differences for a number of these properties. Next, using the two-way ANOVA and tree classifier statistical approaches we assessed the physiological heterogeneity of FS interneurons as a function of cell type (ChCs vs. BCs) and species (monkey vs. rat). A number of ChCs-BCs differences (including both those previously reported and some newly revealed in this study) were conserved across species (including fast and medium afterhyperpolarization, firing frequency, and depolarizing sag), whereas the first spike latency was found to be a species-specific property. We conclude that certain intrinsic membrane properties can be used for the electrophysiological identification of ChCs and BCs in different species and that the contribution of each cell type to cortical network functions may differ across species.

## Methods

### Slice preparation

Brain slices were obtained from adult (56–135 days, 350–550 g; n = 20) male Wistar rats and young adult (4–5 yr old; 3.5–6.0 kg; n = 15) male long-tailed macaque monkeys (*Macaca fascicularis*). All animals were treated in accordance with the guidelines outlined in the National Institutes of Health Guide for the Care and Use of Laboratory Animals and approved by the University of Pittsburgh Institutional Animal Care and Use Committee (protocols ## 0207751A-1, 0507655, 0504220). Rats were deeply anesthetized with halothane and decapitated. The brain was quickly removed and immersed in ice-cold pre-oxygenated artificial cerebrospinal fluid (ACSF). Tissue blocks containing the prelimbic cortex in rats were excised for slicing. The protocol used to obtain brain tissue blocks from monkey PFC was described previously [16]. Prior to any surgical manipulations, the monkeys were either cage-housed or pen-housed, alone or as a pair depending on availability and compatibility of cage-mates. Following surgery animals were single housed in cages. Animals were fed a standard diet of dry biscuits (Lab Diet Monkey Diet, PMI Nutrition International, Brentwood, MO), and a variety of fresh fruit or vegetables daily. They are also provided a foraging mixture of seeds, nuts and corn in their bedding each morning. All animals are provided with a rotation of novel toys and manipulanda both inside and outside their pens or cages and every room has either a DVD player or radio with changing programming throughout the week. Monkeys requiring single housing were put on an enhanced enrichment schedule with increased foraging opportunities, human interactions, stimulation of all five senses, and means to control their environment though manipulations and cognitive stimulating activities. For the surgery, monkeys were treated with ketamine hydrochloride (25 mg/kg im), dexamethasone phosphate (0.5 mg/kg im), and atropine sulfate (0.05 mg/kg sc). Endotracheal anesthesia was maintained with 1% halothane in 28% O_2_-air. For the terminal anesthesia, animal were given an overdose of pentobarbital (30 mg/kg) and were perfused through the heart with ice-cold modified ACSF. All efforts were made to minimize suffering in both rats and monkeys. Coronal slices (350 µm thick) were cut with a vibratome (Model VT1000S, Leica, Nussloch, Germany). Slices were incubated at 37°C for 0.5–1 h and further stored at room temperature until transfer to a recording chamber perfused with ACSF at 31–32°C. The recording temperatures were identical for both species. Through all steps of the experiments, ACSF of the following composition was used (in mM): 126 NaCl, 2.5 KCl, 1.25 NaH2PO4, 1 MgSO4, 2 CaCl2, 24 NaHCO3, and 10–20 dextrose. ACSF was perfused with 95% O_2_-5% CO_2_ gas mixture.

Some of the electrophysiological parameters from all monkey and rat FS BCs and from all monkey ChCs were published previously [18], [20]. Here, in addition, a new critical electrophysiological parameter, medium afterhyperpolarization (mAHP) amplitude, was measured in the recordings from the interneurons. Also, in this study, for the first time, we compared electrophysiological parameters using the two-way ANOVA and tree classifier analyses in order to delineate significant species-specific and species-independent differences between BCs and ChCs. None of the data from the rat ChCs have been reported previously.

### Electrophysiological recordings

Whole cell voltage recordings were made from layer 2/3 neurons visualized by infrared differential interference contrast videomicroscopy using a Zeiss Axioskop 2 FS microscope, equipped with a 40 water-immersion objective and a Dage-MTI NC-70 video camera (Dage-MTI Television, Michigan City, IN). Interneurons were identified based on their round or oval cell body and lack of apical dendrite. Patch electrodes were filled with an internal solution containing (in mM): 114 K-gluconate, 6 KCl, 10 HEPES, 4 ATP-Mg, and 0.3 GTP; pH was adjusted to 7.25 with KOH. Biocytin (0.5%; Molecular Probes, Eugene, OR) was added to the solution for later morphological identification of the recorded neurons. Electrodes had 5- to 12-MΩ open-tip resistance. Voltages were amplified with an IE-210 electrometer (Warner Instruments, Hamden, CT) or a Multi-Clamp 700A amplifier (Axon Instruments, Union City, CA) operating in bridge-balance mode. Signals were filtered at 5 or 4 kHz in the IE-210 and the MultiClamp, respectively, and acquired at a sampling rate of 20 kHz using a 16-bit-resolution Power 1401 interface and Signal software (CED, Cambridge, UK). Access resistance and capacitance were compensated on-line. Access resistance typically was 15–30 MΩ and remained relatively stable during experiments (±30% increase) for the cells included in the analysis. Membrane potential was not corrected for the liquid junction potential. Recordings of the electrophysiological membrane properties were performed in the absence of synaptic blockers.

### Electrophysiological data analysis

To characterize the membrane properties of neurons, hyper- and depolarizing current steps were applied for 500 ms in 5- to 10-pA increments at 0.5 Hz. Input resistance (Rin) was measured from the slope of a linear regression fit to the voltage-current relation in a hyperpolarizing range relative to the resting membrane potential (RMP). The membrane time constant was determined by single-exponential fitting to the average voltage responses activated by hyperpolarizing current steps (5–15 pA). Sag was estimated at the hyperpolarizing current steps as the difference between the most negative membrane potential and the membrane potential at the end of the step as the percentage relative to the voltage deflection from the RMP at the end of the sweep. Importantly, the sag can be affected by the RMP as well as by the magnitude of the voltage deflection produced by the hyperpolarizing current pulses. To make the measures of sag comparable across groups, only the cells with similar RMP values were included in the analysis of the sag. The average RMP in the four groups after selection was: in monkey ChCs −67±5.3 mV; BSs −67±3.5 mV; in rat ChCs −67±4.3 mV; BSs −67±3.8 mV. In addition, the sag was measured on the sweeps with the maximum voltage deflection within the range of −11÷−12 mV.

A series of depolarizing current steps of gradually increasing amplitude were used to evoke action potentials (AP). All AP measures were taken from the first AP of the first sweep that reached the spike threshold. Peak amplitudes of the AP and the fast afterhyperpolarization (fAHP) amplitude were measured relative to the AP threshold (level of voltage deflection exceeding 10 mV/1 ms). Duration of the AP was measured at its half-amplitude. mAHP amplitude was measured after the depolarizing responses as the most negative voltage deflection relative to the RMP. Frequency was estimated at the level of 60 pA above rheobase as a reciprocal of average interspike interval within the last 250 ms of depolarizing response. Adaptation ratio (AR) coefficient was used to describe spike frequency adaptation in spike trains. First, the ratio between the first and the last interspike interval was calculated for the each stimulation current intensity. Then, the AR coefficient was estimated from the linear regression of AR versus current at 60 pA above rheobase. The level of 60 pA above rheobase was chosen for estimation of frequency and AR coefficient since it was the lowest stimulation current intensity to produce relatively regular firing in rat FS BCs with very few quiescent periods [20].

Cells were identified as FS based on the results of the previously performed cluster analysis, ANOVA, and Fisher's least significant difference post hoc test [17]. The parameters with the most discriminative values, action potential duration (AP duration; average value 0.37±0.09 ms), and AR coefficient (average value 0.82±0.21) were used as criteria for FS interneurons: “AP duration +1.5 SD” as the high limit and “AR coefficient – SD” as the low limit. Accordingly, only cells with the spike half-duration <0.51 ms and AR coefficient >0.61 were included in the analysis. The aforementioned criteria correspond to those previously used in the neocortex of young rats [4] and adult rats [21].

### Morphological data analysis

To identify cell morphology after the electrophysiological experiments, neurons were filled with biocytin (0.5%) added to the pipette solution. After recordings, slices were immersed in 4% paraformaldehyde in 0.1 M phosphate-buffered saline (PBS) and then were kept in storing solution (equal parts of glycerol, ethylene glycol, and 0.1 M PBS) at −80°C. In some cells biocytin was visualized with streptavidin-Alexa Fluor 633 conjugate (for details see Zaitsev et al., 2009). Briefly, slices were incubated with streptavidin-Alexa Fluor 633 conjugate (dilution 1∶500; Invitrogen) for 24–48 h at 4°C in in PBS containing 0.4% Triton X-100). After this, cells were confocally reconstructed for morphological identification using an Olympus FluoviewTM 500 confocal laser scanning microscope (Olympus America, Melville, NY). Slices from other experiments were resectioned at 40–50 µm. The sections were treated with 1% H_2_O_2_ for 2–3 h at room temperature, rinsed, and incubated with the avidin-biotin-peroxidase complex (1∶100; Vector Laboratories, Burlingame, CA) in PBS for 4 h. Sections were rinsed, stained with Ni-3,3-diaminobenzidine (DAB), mounted on gelatin coated glass slides, dehydrated, and coverslipped. Cells were morphologically identified as BCs or ChCs based on the confocal reconstructions or/and development of biocytin. Some cells were three-dimensionally reconstructed using the Neurolucida neuron tracing system with NeuroExplorer software (MBF Bioscience, Williston, VT).

### Statistical analysis

Two-tailed *t*-tests were used for group comparisons in most cases. Unless otherwise noted, values are presented as means ± SD. To examine cell type-specific and species-specific differences in membrane properties, two-way ANOVAs were performed. To delineate the electrophysiological membrane properties that could predict the morphology of FS interneurons (either “basket” or “chandelier”), the tree classifier analysis was performed with estimation of global cross validity (CV) cost (Breiman, 1984). In global cross-validation, the entire analysis was replicated a specified number of times (usually 3 times) holding out a fraction of the learning sample equal to 1 over the specified number of times, and using each hold-out sample in turn as a test sample to cross-validate the selected classification tree. The CV costs computed for each of the test samples was then averaged to give the estimation of the global CV costs. The more correctly the test samples were classified the lower the global CV cost was. Statistical tests were performed using Excel (Microsoft, Redmond, WA) or Statistica 8 (Statsoft, Tulsa, OK).

## Results

### Identification of ChCs and BCs with FS phenotype

In our previous publications, we successfully employed different approaches to delineate groups of interneurons in monkey PFC, including morphological features [18], [22], electrophysiological membrane properties [16], [17], or neurochemical content [6], [23]. In the present study, we addressed the physiological heterogeneity of interneurons with a FS phenotype in rat and monkey PFC. We predicted that this heterogeneity is accounted for, at least in part, by differences between BCs and ChCs. To test this prediction, we selected from our library of recordings all of the cells that satisfied electrophysiological criteria for the FS phenotype and that were clearly identified morphologically as either ChCs or BCs.

**Figure 1:**
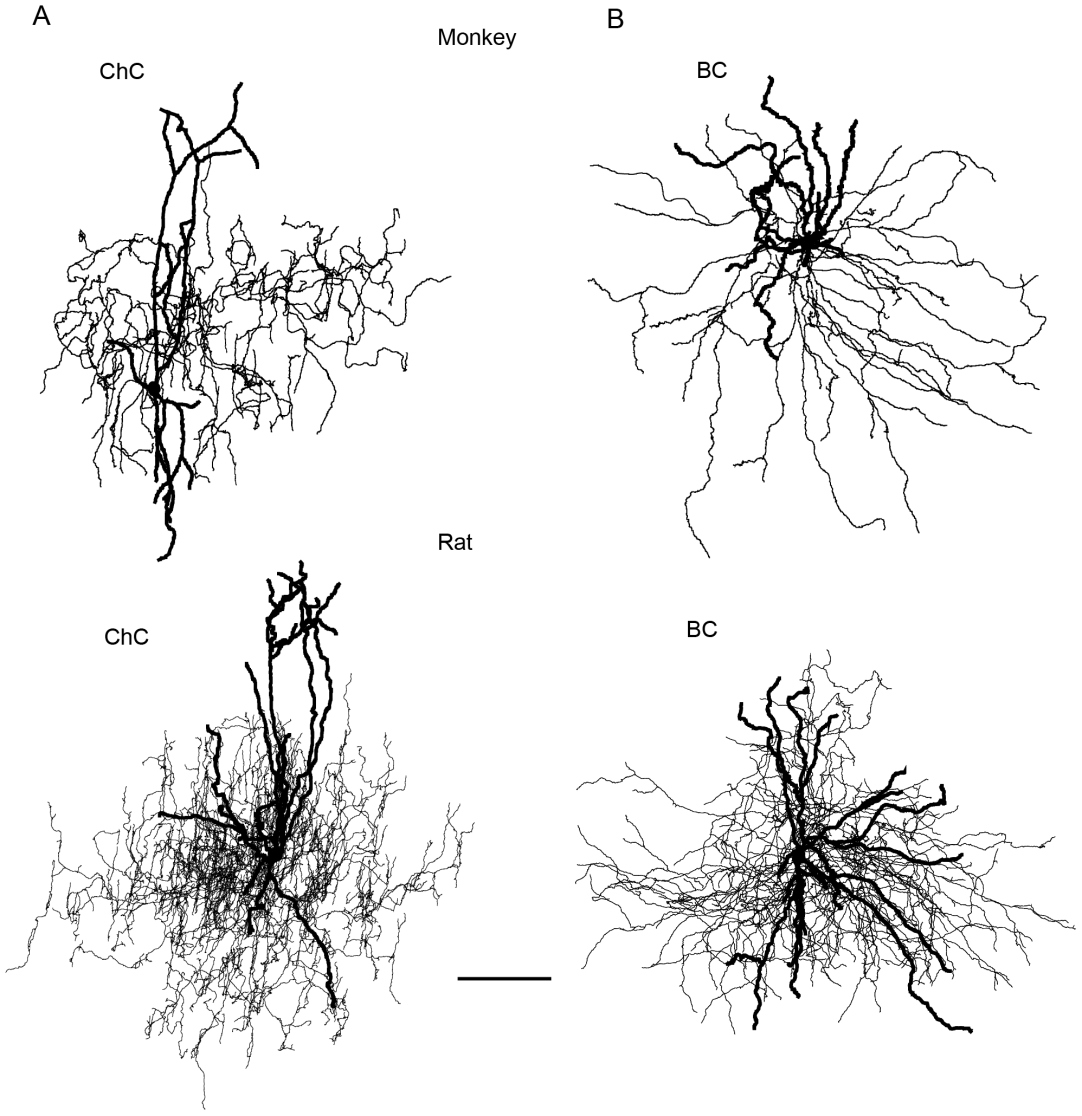
Morphological features of ChCs and BCs in monkey and rat. A. Representative examples of monkey and rat ChCs (note vertical axonal branches that contain arrays of cartridges). B. Representative examples of monkey and rat BCs (note axonal branches going both horizontally and vertically) (scale bar 100 µm).

Cells were identified as FS based on the results of previously performed cluster analysis, ANOVA, and Fisher least significant difference post hoc test [17]. Only BCs with spike half-duration <0.51 ms (averaged AP duration plus 1.5 SD) and AR coefficient >0.61 (averaged AR coefficient minus SD) were included in the analysis (See Methods). These FS cells were identified either as ChCs, or BCs based on their morphological features. The morphology of ChCs have been described in various studies from rat [4] and monkey [18], [22] neocortex. In this study, both rat and monkey ChCs had characteristic vertical arrays of axonal boutons (cartridges) and smooth multipolar dendrites (Figure 1A). Their dendritic and axonal trees were confined mostly to layers 2–3. FS interneurons from monkey and rat PFC were classified as “basket” based on previously described morphological features [4], [22]. BCs somata had a round or vertical oval shape and were located in layers 2/3. The cells possessed smooth and multipolar dendrites (Figure 1B). The axon of the cells originated from the cell body or one of the primary dendrites. Axons spread either in all directions or predominantly horizontally and located predominantly within layers 2–3.

### Physiological heterogeneity of FS interneurons across species

We first addressed the contribution of species identity (monkey vs. rat) to the physiological variability of FS interneurons. Previously, we reported a number of differences in electrophysiological membrane properties of BCs from rat and monkey PFC [20]; the present analyses mostly confirmed these findings and also revealed an important difference in mAHP amplitude between rat and monkey BCs (Table 1).

**Table 1:** Membrane properties of ChCs and BCs in rat and monkey PFC.

	t-test				ANOVA analysis					
	Monkey		Rat		species		cell type		species* cell type	
	ChCs (n = 13)	BCs (n = 39)	ChCs (n = 9)	BCs (n = 31)	F_1,88_	p	F_1,88_	p	F_1,88_	p
RMP, mV	−65±8	−68±8	−68±5	−67±6	0.04	0.85	0.17	0.68	2.0	0.16
Rin, MΩ	321±114∧∧∧	251±130∧	167±58	182±83	**18**	**0.00**	1.1	0.31	2.6	0.11
Time constant, ms	10±3	9±3∧∧	8±2	7±3	**8.1**	**0.01**	1.4	0.25	0.05	0.83
Sag, %	24±7** ∧∧∧ (n = 8)	16±10∧∧∧ (n = 19)	10±2*** (n = 8)	0±1 (n = 23)	**63** (F_1,54_)	**0.00**	**23** (F_1,54_)	**0.00**	0.34 (F_1,54_)	0.56
Rheobase, pA	40±27** ∧∧∧	75±48∧∧∧	108±23	123±58	**23**	**0.00**	**5.2**	**0.03**	0.14	0.71
1^st^ AP latency, ms	145±174∧	74±68∧∧∧	37±17***	223±152	0.49	0.48	**3.8**	**0.05**	**19**	**0.00**
AP threshold, mV	−43±5∧∧	−41±5∧∧∧	−38±3**	−34±2	**34**	**0.00**	**8.1**	**0.01**	0.61	0.44
AP amplitude, mV	53±12	55±11	60±7*	53±8	1.1	0.29	1.1	0.30	2.9	0.09
AP duration, ms	0.32±0.06	0.34±0.06∧	0.32±0.02***	0.38±0.08	0.64	0.42	**6.5**	**0.01**	0.16	0.69
fAHP amplitude, mV	19±2*	23±8	19±1***	23±5	0.00	1.0	**7.7**	**0.01**	0.04	0.85
mAHP amplitude, mV	7.1±2.0** ∧∧∧	5.0±1.6∧∧∧	4.1±0.7***	0.7±0.6	**117**	**0.00**	**65**	**0.00**	3.6	0.06
Frequency, Hz	105±38*** ∧∧	58±18∧	82±6***	49±15	**11**	**0.00**	**62.6**	**0.00**	1.90	0.17
AR coefficient	0.86±0.19	0.90±0.13	0.75±0.150	0.85±0.12	**5.4**	**0.02**	**4.1**	**0.05**	0.92	0.34

*/**/*** Significantly different between ChCs and BCs within the same species (p<0.05/0.01/0.001).

^∧^/^∧^^∧^/^∧^^∧^^∧^Significantly different between monkey and rat ChCs or between monkey and rat BCs (p<0.05/0.01/0.001).

Bold font: Significantly different at p<0.05.

#### Membrane properties of ChCs in monkey and rat PFC

**Figure 2:**
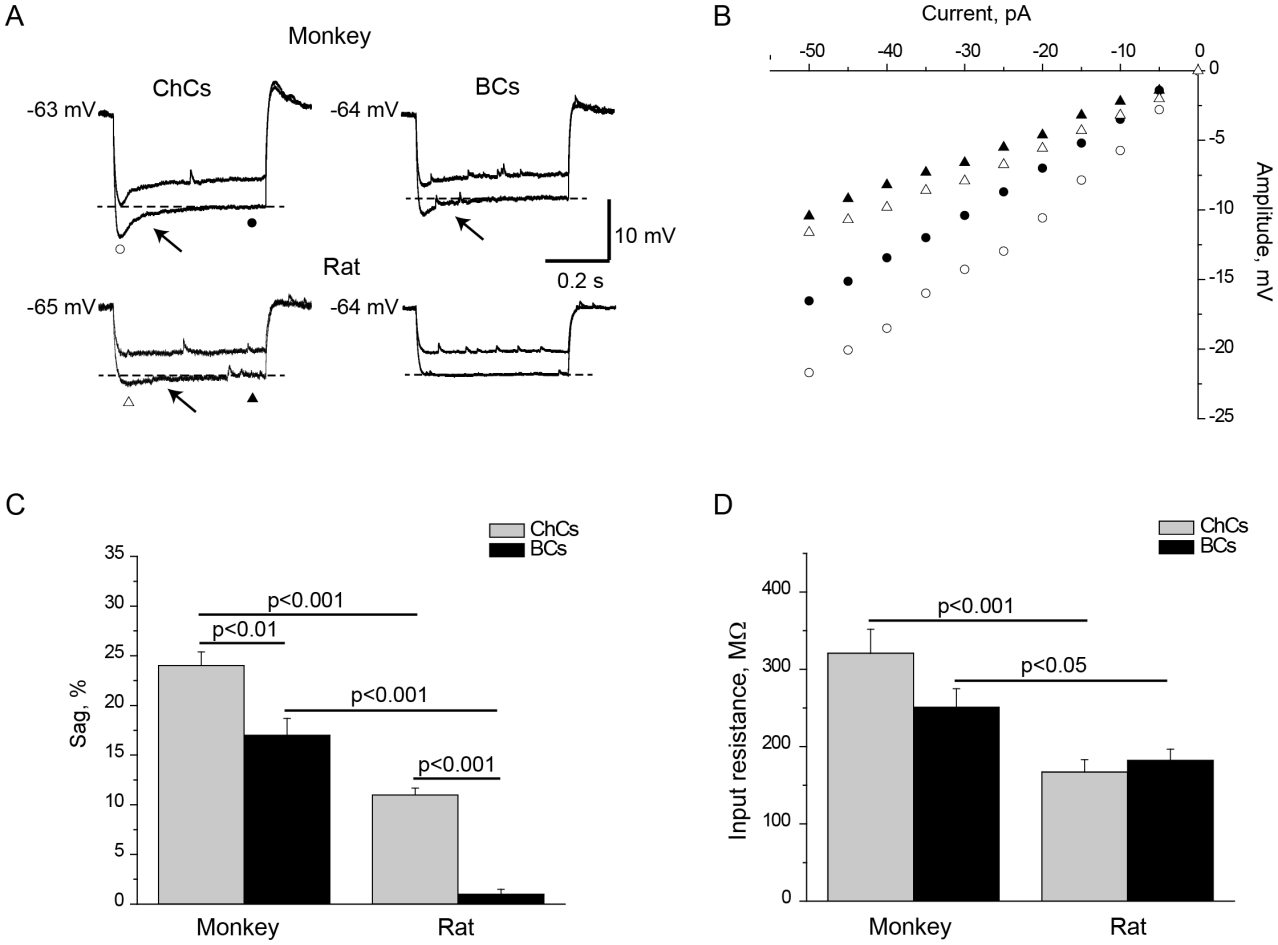
Differences in responses to hyperpolarizing current pulses in ChCs and BCs from monkey and rat. A. Voltage responses to the hyperpolarizing current steps in ChCs and BCs from monkey and rat. Both monkey ChCs and BCs as well as rat ChCs showed time-independent inward rectification (“sag”; arrows). B. Current-voltage plots for traces shown in A. Comparison of average values of sag (C) and Rin (D) in monkey and rat ChCs and BCs. Error bars represent SE.

**Figure 3:**
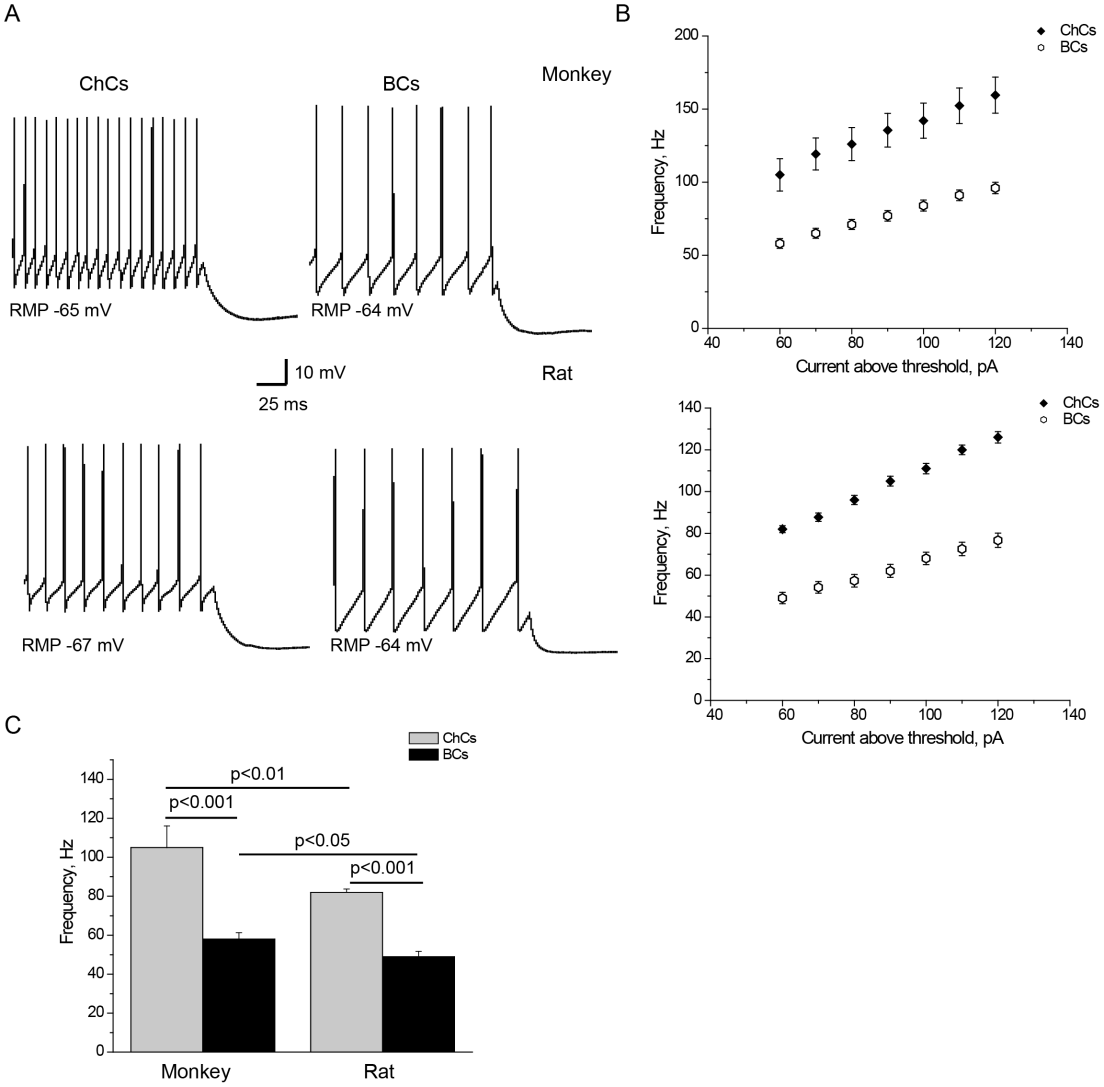
Firing frequency in ChCs and BCs from monkey and rat. A. Representative firing patterns produced by the 60 pA above threshold stimulation current intensity in monkey and rat ChCs and BCs. B. Quantification of population data for firing frequency at different stimulation current intensities in ChCs and BCs from rat and monkey. ChCs fired at higher frequency than BCs at all current intensities. C. Firing frequency was higher in ChCs vs. BCs in both species, as well as in monkey vs. rat in both ChCs and BCs. Error bars represent SE.

**Figure 4:**
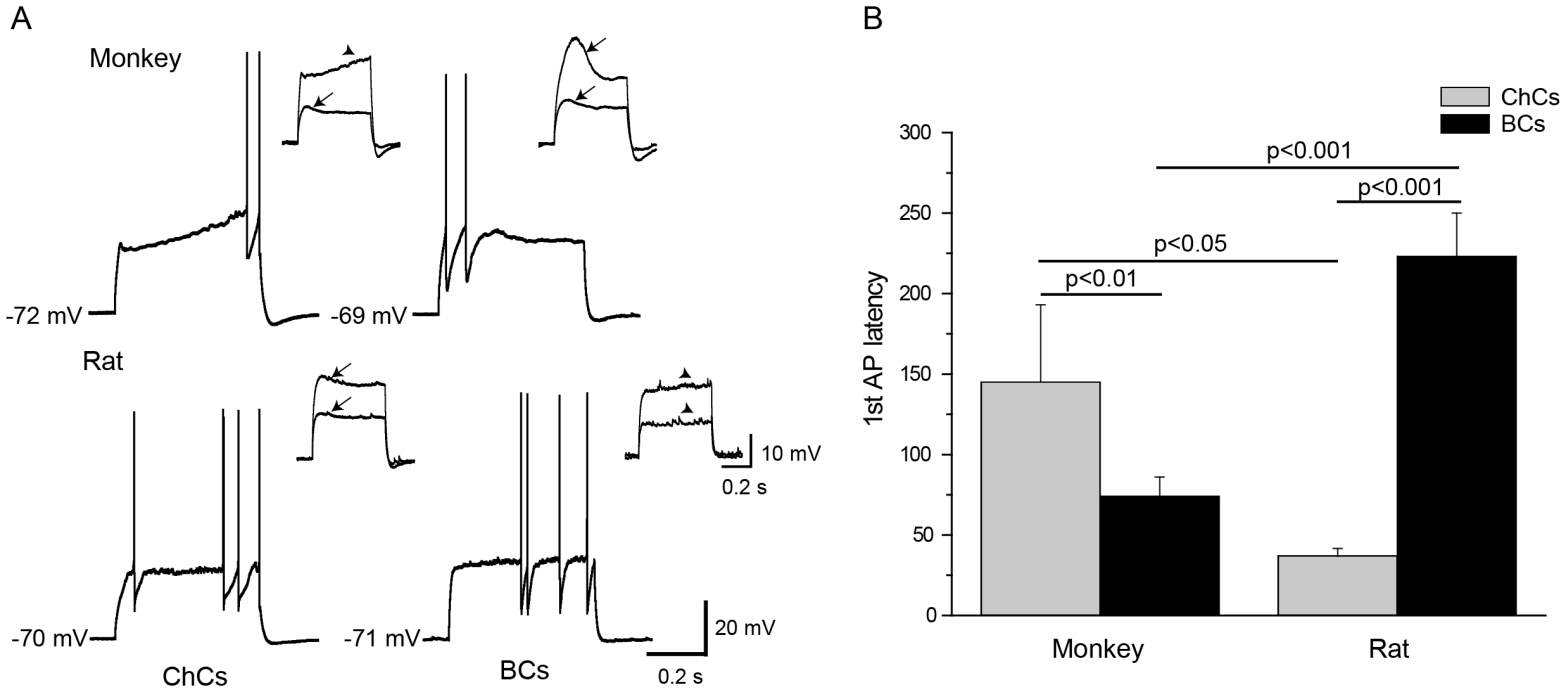
ChCs and BCs differences in the 1^st^ AP latency. A. Representative traces with the first AP in in monkey and rat ChCs and BCs. ChCs but not BCs showed delayed 1st AP/depolarizing ramp at near-threshold levels of stimulation currents in monkey PFC. On the contrary, in rat, BCs but not ChCs showed delayed 1^st^ AP/depolarizing ramp at the near-threshold levels of stimulation currents. Insets: BCs but not ChCs demonstrate hump (arrow) at the sweeps just below firing threshold in monkey PFC. Ramp (arrowhead) is observed in a number of chandelier cells. On the contrary, in rat PFC, ChCs but not BCs demonstrate hump at the subthreshold sweeps. Flattened response (arrowhead) is observed in BCs but not ChCs from rat. *B*. Quantification of population data for the 1^st^ AP latency. Note different direction of ChCs-BCs differences in monkey and rat. Error bars represent SE.

**Figure 5:**
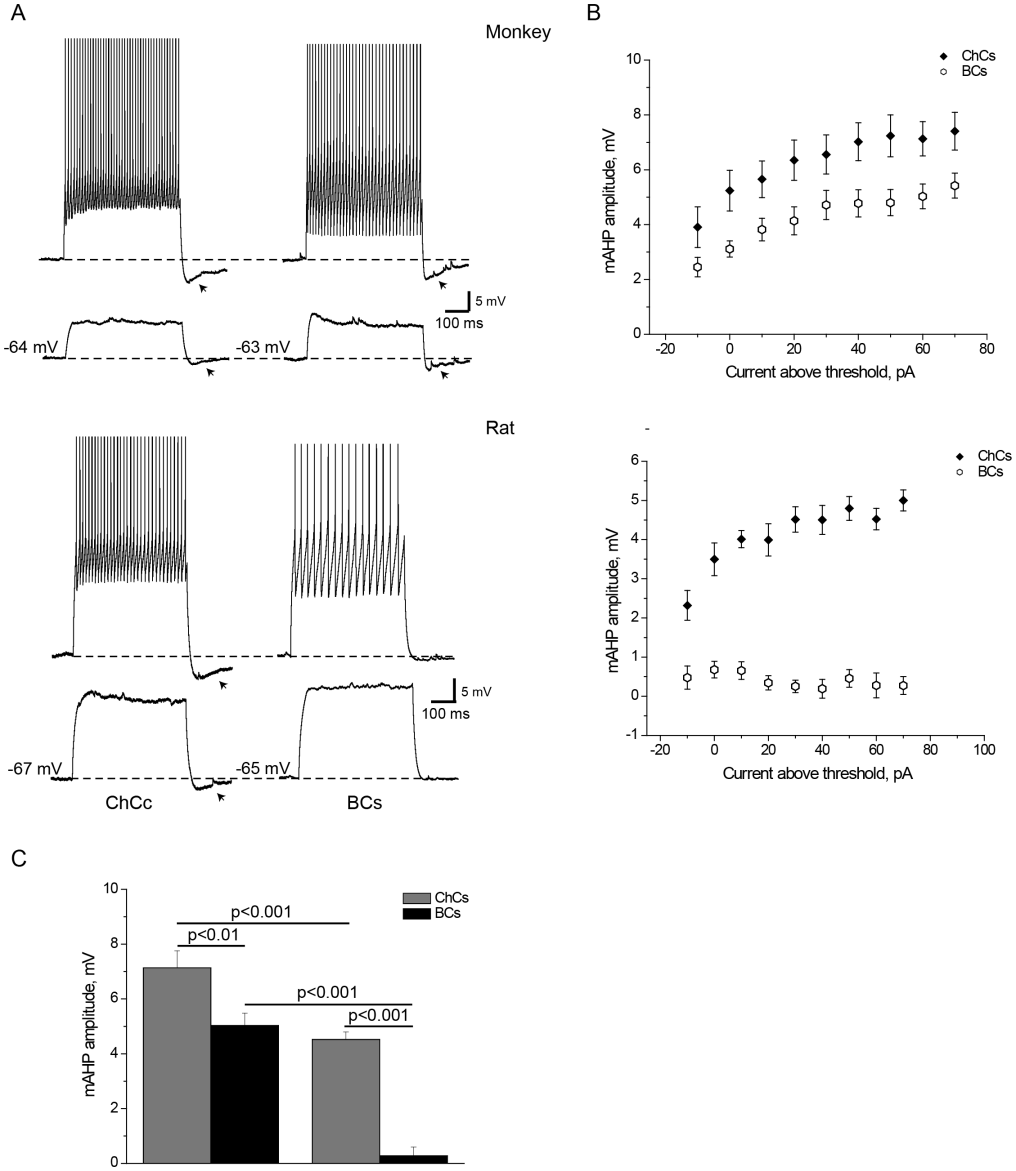
ChCs and BCs differences in mAHP amplitude in rat and monkey. A. Representative traces with sub-and suprathreshold responses to depolarizing current pulses in monkey and rat ChCs and BCs. Arrowheads mark mAHP in monkey ChCs and BCs, as well as in rat ChCs. Note that in rat, mAHP was observed in ChCs, and was almost absent in BCs, while in monkey, mAHP could be observed in both cell types, but was more pronounced in ChCs than in BCs for the same current intensities above rheobase. B. Quantification of population data for mAHP amplitude at different stimulation current intensities. X-axis values: “0 pA” = rheobase, “−10 pA” = current intensity 10 pA below rheobase, all positive values = current above rheobase. C. mAHP amplitude at the stimulation level of 60 pA above rheobase was larger in ChCs than BCs in both species, as well as in monkey than in rat for both ChCs and BCs. Error bars represent SE.

Comparison of electrophysiological membrane properties of ChCs from monkey and rat PFC revealed species differences. The ChCs membrane properties that differed between the two species included Rin, rheobase, frequency, 1^st^ AP latency, AP threshold, sag and mAHP amplitude (Table 1). Rin was substantially higher in monkey than in rat ChCs (321±114 vs. 167±58 mΩ, p<0.001) (Figure 2), whereas the rheobase was lower in the former (40±27 vs. 108±23 pA, p<0.001). Firing frequency was considerably higher in monkey ChCs (105±38 Hz) than in rat (82±6 Hz, p<0.01) (Figure 3). Monkey ChCs fired the first AP with a longer latency (145±174 ms) than rat ChCs (37±17 ms, p<0.05) (Figure 4). Surprisingly, this difference was the opposite of the species difference observed for BCs where the 1^st^ AP latency was longer in rat than in monkey [20]. AP threshold was more negative in monkey ChCs than in rat. Sag and mAHP amplitude were more pronounced in monkey as compared to rat ChCs (Figure 2C, 5C, Table 1).

#### Interaction between “species” and “cell type” through a two-way ANOVA

In order to determine, first, how species differences contribute to the physiological heterogeneity of FS interneurons and, second, how they interact with the morphological cell-type factor, we used a two-way ANOVA. The results of this analysis suggest that species had a major effect on the properties of FS interneurons. Indeed, according to ANOVA analysis the species factor had significant F_1,88_ values (ranging from 5.4 to 117) for 8 out of 13 electrophysiological parameters including Rin, rheobase, time constant, sag, AP threshold, mAHP amplitude, firing frequency and AR coefficient, although the differences in time constant between monkey and rat ChCs as well as between monkey and rat AR coefficient for both cell types did not achieve statistical significance.

Interestingly, the 1^st^ AP latency was different between monkey and rat for both ChCs and BCs. Also, for this variable, there was significant interaction between species and cell-type factors (F_1,88_ = 19, p<0.00; Table 1). Indeed, this parameter showed species differences in the opposite directions: the 1^st^ AP latency was longer in monkey ChCs than in those from rat, whereas in monkey BCs it was shorter than in rat.

Therefore, these analyses demonstrated an overall difference between monkey and rat FS interneurons, and that the “species differences” for 8 out of 13 electrophysiological membrane properties were similar for both morphological cell types, whereas for 1 out of 13 properties, these differences were in the opposite direction for ChCs and BCs.

### Physiological heterogeneity of FS interneurons defined by morphological cell type

Next we addressed how the morphological cell type factor defines physiological variability of FS interneurons. Differences in electrophysiological membrane properties between ChCs and BCs were assessed by the t-test separately for monkey and rat and by the two-way ANOVA analysis for the whole population of cells. Thus, we were able to define whether these “cell-type differences” were similar in monkey and rat. Interestingly, although some ChCs-BCs differences were similar in both species (species–independent), others were species-specific.

#### Species-independent ChCs-BCs differences

**Figure 6:**
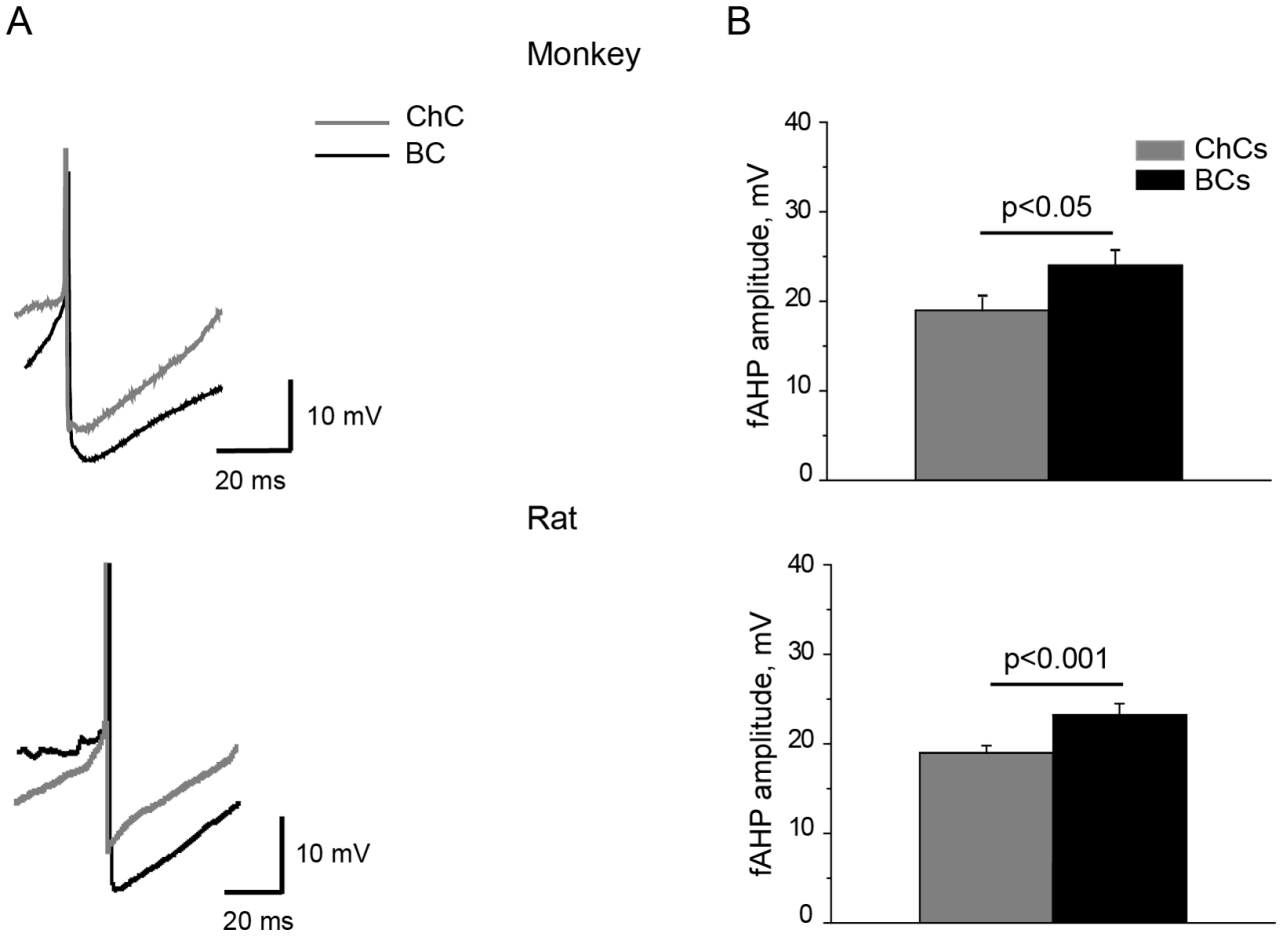
ChCs and BCs differences in fAHP amplitude in both species. A. Action potential (truncated) produced by depolarizing current pulses are followed by fAHP in monkey and rat ChCs (gray) and BCs (black). *B*. Quantification of population data for fAHP amplitude in monkey and rat ChCs (gray) and BCs (black). In both monkey and rat, ChCs have smaller fAHP amplitude than BCs. Error bars represent SE.

We found that sag, mAHP amplitude, fAHP amplitude and firing frequency differed substantially between ChCs and BCs in both monkey and rat (Table 1, Figure 2, 3, 5, 6). Each of these characteristics also showed cell-type specificity in the ANOVA for the pooled cell population. For example, firing frequency was substantially higher in ChCs than in BCs in both monkey (105±38 Hz vs. 58±18 Hz, p<0.001) and rat (82±6 vs. 49±15, p<0.001) with “cell-type” factor of F_1,88_ = 62.6, p>0.001 in ANOVA (Table 1, Figure 3). Importantly, although firing frequency, sag and mAHP amplitude also demonstrated interspecies differences, there was no interaction between the two factors (p>0.05 for “species*cell type”), indicating that cell-type differences for those properties exist independently of their species identity. For example, for frequency, “species*cell type” F_1,88_ was 1.9, p = 0.17. Indeed, firing frequency was higher in ChCs than in BCs in both monkey and rat, i.e. this difference was species-independent.

According to the two-way ANOVA analysis, mAHP amplitude had the largest F-value (F_1,88_ = 65, p<0.001) for the “cell type” factor relative to the all other properties. Indeed, in rat PFC, the most striking ChCs-BCs difference was in the amplitude of the mAHP that followed responses to rectangular depolarizing current pulses. This AHP could be observed when the responses were still subthreshold and increased with the increase in the stimulation current (Figure 5). With high stimulation currents exceeding rheobase, this mAHP followed the trains of action potentials. While ChCs demonstrated a pronounced mAHP with the average amplitude of 4.1±0.7 mV with the stimulation current of 60 pA above rheobase, BCs had a barely visible mAHP with average amplitude that never exceeded 0.7 mV (Figure 5A and B). Unlike ChCs, BCs did not show a substantial increase in the mAHP amplitude with increasing stimulation current. Individual ChCs and BCs never had overlapping values of the mAHP amplitude for the stimulation current intensities that exceeded the level of 10 pA below rheobase. All the aforementioned differences between ChCs and BCs in the depolarization-induced mAHP amplitude makes it a reliable criterion to distinguish the two cell types in rat PFC. In monkey PFC, the mAHP showed a substantial increase in amplitude with the increase in stimulation current (Figure 5). Although it was well pronounced in both ChCs and BCs, its amplitude was larger in ChCs than in BCs for stimulation currents above rheobase (Figure 5B).

Sag measured on the responses to the hyperpolarizing current pulses had the third largest two-way ANOVA F-value (F_1,54_ = 23, p<0.001), which indicates its importance for the electrophysiological classification. Sag was more pronounced in ChCs as compared to BCs in both monkey and rat (Figure 2, Table 1).

Amplitude of fAHP was larger in BCs as compared to ChCs in both monkey and rat (Figure 6, Table 1). Two-way ANOVA revealed that ChCs-BCs differences in fAHP amplitude were defined by the “cell type” factor (F_1,88_ = 7.7, p<0.01), but not by the “species” factor, unlike mAHP amplitude, sag and firing frequency that show not only cell-type differences but also species differences.

Three electrophysiological membrane properties: AP threshold, AP duration and rheobase demonstrated significant values for the “cell type” factor according to the two-way ANOVA (Table 1). Separate comparison of AP threshold and AP duration revealed cell type difference in rat, while only similar tendencies were observed in monkey (AP threshold had a tendency to be lower (p = 0.17) and AP duration had a tendency is to be shorter (p = 0.08) in ChCs than in BCs). The rheobase was significantly lower in ChCs than in BCs in monkey with the similar tendency in rat (Table 1). AP threshold and rheobase were also influenced by the species factor.

Although firing in FS interneurons is generally considered non-adapting, some adaptation can take place making the last interspike interval slightly longer than the first one, especially with longer current pulses [24]. Interestingly, t-test comparisons between cell types for both species did not reach the level of significance (Table 1), and yet, according to the two-way ANOVA, the AR had a significant values of “cell type” factor (Table 1) since in both monkey and rat, ChCs tend to have more adaptation of firing frequency as compared to BCs.

#### Species-specific ChCs-BCs differences

One of the species-specific ChCs-BCs differences was the 1^st^ AP latency. According to the two-way ANOVA, this parameter had the strongest interaction between cell-type and species factors, indicating that the cell type differences in the 1^st^ AP latency are dependent on species. In monkey PFC, many ChCs started firing with a substantial delay: 6 out of 13 cells had the latency of the 1^st^ AP>100 ms (Figure 4). In contrast, the majority of BCs had a short latency of the 1^st^ AP. Surprisingly, in rat PFC, ChCs and BCs differed in their 1^st^ AP latency in the opposite direction. While all the ChCs always fired the first spike with a relatively short latency, the majority of the BCs demonstrated a substantially delayed first spike: about 70% of BCs had the 1^st^ AP latency >100 ms (Figure 4A and B).

Interestingly, in rat, ChCs exhibited a subthreshold membrane potential response to depolarizing current pulses with a slight hump, whereas BCs had a depolarizing response that looked either flat, or had a slight ramp (Figure 4A). Curvy response (“hump”) prior to the AP onset was described previously in ChCs, but not in BCs in mouse neocortex [10]. In contrast, in monkey PFC, the majority of BCs (31 out of 39) demonstrated a hump of more than 15% (see Methods) on the sweep that was followed by the sweeps with APs. At the same time, the majority of ChCs (11 out of 13) did not show a hump on the depolarizing sweep produced by the current just below the rheobase. Moreover, some of them (n = 4) generated a depolarizing ramp (Figure 4A). Lack of a hump on the subthreshold depolarizing responses, and the appearance of a ramp-like structure would lessen the probability of generating a spike at the beginning of the sweep with a short latency, and lead to generation of a delayed spike.

### Membrane properties for electrophysiological identification of ChCs and BCs

To delineate the electrophysiological membrane properties that can be effectively used to identify ChCs and BCs, the tree classifier method was used [25]. Classification trees are used to predict membership of cases or objects in the classes of a categorical dependent variable from their measurements on one or more predictor variables. Here, electrophysiological membrane properties have been used as such predictor variables to make hierarchical decisions whether a cell can be identified as ChC or BC. Since substantial interspecies differences were detected for 8 of 13 parameters used for ChCs-BCs comparison, classification trees were built separately for the cells from rat and monkey PFC.

**Figure 7:**
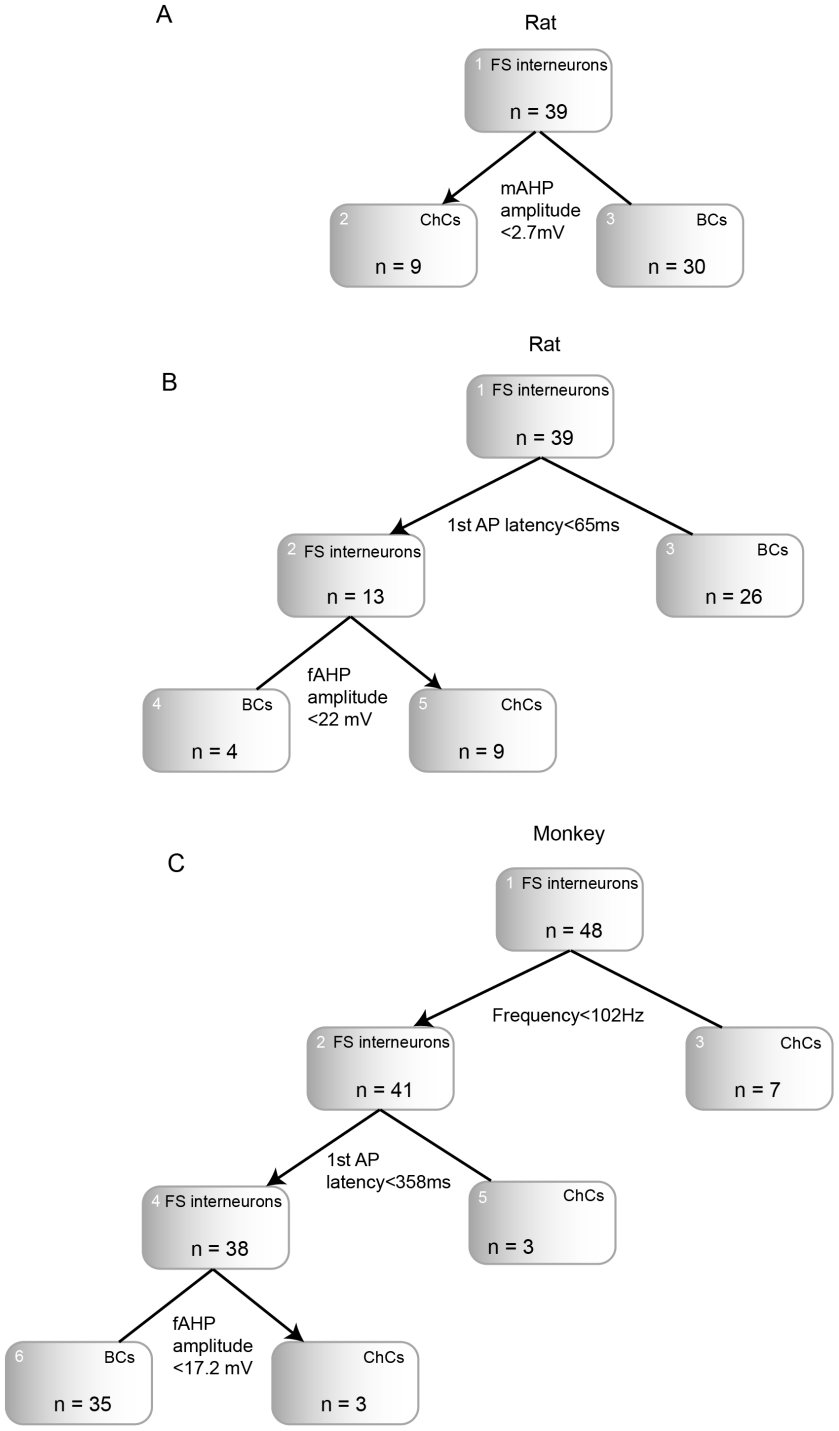
Classification trees for monkey and rat FS interneurons. A. Classification tree for rat FS interneurons based on mAHP amplitude. B. Classification tree for rat FS interneurons based on 1^st^ AP latency and fAHP amplitude. C. Classification tree for monkey FS interneurons based on frequency, 1^st^ AP latency and fAHP amplitude. Arrow indicates the box to follow if the condition is “True”.

In rat, the classification tree analysis defined the mAHP amplitude as the most important property for ChCs-BCs differences. It was followed by the 1^st^ AP latency and fAHP amplitude (Figure 7). However, during the classification tree construction process we found that the mAHP amplitude was overwhelmingly dominant and could be used by itself to construct an accurate classification tree: all rat FS interneurons with mAHP amplitude ≥2.65 mV are ChCs, whereas the remaining FS cells are BCs (Figure 7A). The two trees classifiers constructed for rat FS interneurons had global cross-validity cost of 0 and 0.2 (see Methods) meaning that the classification tree based on mAHP amplitude was the most effective in dividing our population into ChCs and BCs. Indeed, it classified our population into the ChCs and BCs types without mistakes. And the estimated CV cost that equals “0”, predicts “0” misclassifications for the other FS cell populations as well. The second tree based on the 1^st^ AP latency and fAHP amplitude misclassified 5 out of 39 cells in the learning sample (2 ChCs and 3 BCs) and predicts 20% of misclassifications (Figure 7B).

In monkey, the tree classifier with the lowest cross-validity (global cross-validity cost = 0.167) included frequency, 1^st^ AP latency and fAHP amplitude (Figure 7C). First, all monkey FS interneurons were classified based on the values of firing frequency: cells with higher frequency (>102 Hz) were identified as ChCs, while cells with the lower frequency were divided based on the 1^st^ AP latency into ChCs (1^st^ AP latency >358 ms) and BCs. The latter group was then divided into ChCs and BCs based on the fAHP amplitude: cell with the fAHP amplitude less than 17 mV were identified as ChCs. This tree misclassified 2 out of 48 monkey FS cells in the learning sample (1 BC was classified as “ChCs” and 1 ChC was classified as “BCs”), and predicts 16.7% mistakes for any other monkey FS cell populations.

## Discussion

In this study, we addressed physiological heterogeneity of FS interneurons as a function of species (monkey vs. rat) and morphology (ChCs vs. BCs). We demonstrated differences between monkey and rat ChCs electrophysiological membrane properties that have not been previously reported, and confirmed our previously reported differences in BCs between monkey and rat BCs [20]. We showed an overall difference between FS neurons of monkey versus rat, and that the species differences for the most of the properties are similar for ChCs and BCs. Next, we assessed morphological cell-type-associated differences in electrophysiological membrane properties. The majority of these properties were different between ChCs and BCs either in one or in both species. Some of these ChCs-BCs differences were “species-independent” while others were “species-specific”. Here, for the first time, we reported a striking difference in mAHP amplitude that was substantially larger in ChC than BCs in both monkey and rat. For rat interneurons, a classification tree built based on mAHP amplitude unequivocally divided interneurons on ChCs and BCs. Another tree was built based the 1^st^ AP latency and fAHP amplitude. In monkey, the classification tree with the lowest cross-validity global cost was built based on frequency, 1^st^ AP latency and fAHP amplitude.

### Species differences in electrophysiological membrane properties of ChCs and BCs

In this study, for the first time, we demonstrated differences in membrane properties between rat and monkey ChCs. The ChCs properties that differed between the two species included Rin, rheobase, sag, AP threshold, 1^st^ AP latency, mAHP amplitude and frequency (Table 1). Also, for the first time, we reported here the important difference in mAHP amplitude between rat and monkey BCs (Table 1).

Here we demonstrated that there is a general difference between FS neurons from rat and monkey PFC, and that the differences for the 8 out of 13 electrophysiological membrane properties are similar for ChCs and BCs, while 1 out of 13 properties is different in rat and monkey in the opposite directions. Interspecies differences in electrophysiological membrane properties for both BCs and ChCs reported here and elsewhere [20], could underlie different spiking behavior of FS units during performance of working memory tasks in monkey and rat [26], [27]. Indeed, in monkey PFC, FS units (putative FS interneurons) fire at a frequency of 40–60 Hz during the delay period of oculomotor delayed response task [27], [28], whereas in rat PFC, prefrontal FS units fire with a lower frequency of around 12 Hz when rats perform a delayed spatial alternation task [26]. These differences in the spiking behavior of putative FS interneurons between monkey and rat PFC can be explained by the differences in their intrinsic membrane properties reported in this study.

It should be noted, that in this study, all recordings of the electrophysiological membrane properties were performed in the absence of synaptic blockers. Previously, it was shown that *in vivo*, spontaneous synaptic activity can increase cellular Rin [29]. Our previous study demonstrated that in rat BCs frequency of miniature postsynaptic potentials is higher than in monkey [20], which could be responsible for possible overestimation of Rin in rat in the absence of synaptic blockers. And yet synaptic blockers did not produce any significant changes in Rin in both monkey and rat BCs [20].

In addition, one of our findings reported here is that ChCs-BCs differences can be species-specific. Thus, we showed that the electrophysiological membrane property such as the 1^st^ AP latency, as well as the shape of the subthreshold responses to the depolarizing current pulse were species-specific (Figure 4). One of the recent studies of ChCs-BCs differences was made in a different species (mouse) from the ones used in this study [10]. In Woodruff et al., the most striking ChCs-BCs differences were differences in Rin, rheobase, time to the 1^st^ AP and membrane time constant [10]. Moreover, the intrinsic electrophysiological properties that separate ChCs and BCs in mouse cortex layer 2 have been proposed to be used to predict the morphology of individual FS neurons [10]. Interestingly, the ChCs-BCs differences in the 1^st^ AP latency reported in Woodruff et al. coincide with those observed in rat but not in monkey in the present study, while the ChCs-BCs differences in rheobase are similar to those reported in monkey. All these data indicate that species identity contributes substantially to physiological heterogeneity of FS interneurons.

Currently, rodents are the dominant animal model for understanding human cortex function and dysfunction in mental illnesses, yet the validity of the rodent model has not been fully established. Although it is generally believed that a canonical cortical circuit is conserved across different mammalian species [30], a number of cellular level differences between rodents and primates have been reported. For example, in the rat frontal cortex PV-positive interneurons are the largest population of inhibitory neurons [31], whereas calretinin-positive interneurons predominate in the PFC of monkeys [23]. In addition, Ca^2+^-binding proteins and neuropeptides are extensively colocalized in interneurons of the rat frontal cortex [5], but not in the monkey dorsolateral PFC [23]. Furthermore, as described here and in our previous study [20], interspecies differences are also present in the electrophysiological properties in ChCs and BCs. In concert, these findings indicate that findings in rodent model systems have to be interpreted with appropriate care regarding their relevance for the role of GABA neurons in human brain function and dysfunction.

### ChCs and BCs differences in physiological properties

Beginning with the first description of the membrane properties of ChCs and BCs in rat neocortex [4], [32], it has been generally believed that ChCs have a FS phenotype that is indistinguishable from FS basket cells. In this study we demonstrated differences between ChCs and BCs for a number of electrophysiological membrane properties.

One of the membrane properties that showed a striking ChCs-BCs difference in the present study was the amplitude of the mAHP generated at the end of depolarizing responses, both subthreshold and suprathreshold. Such ChCs-BCs difference in mAHP amplitude was never reported before. In rat, the mAHP amplitude allowed for unequivocal ChCs and BCs differentiation. The channels that might be responsible for the mAHP are Ca^2+^-dependent K^+^ channels, mostly small conductance apamin-sensitive K^+^ channels [33]. These channels could also define firing pattern adaptation. In addition, it was demonstrated that this hyperpolarization was due to the activation of a K^+^ current activated by Na^+^ [34]. In this study, we also report differences in fAHP amplitude between ChCs and BCs in both rat and monkey, which can potentially reflect differences in delayed rectifier Kv3 channels with very fast deactivating kinetic [35].

Previously, we demonstrated differences in hyperpolarizing “sag”, or time-dependent inward rectification between ChCs and arbor cells (presumably, FS BCs) from monkey PFC [18]. In this study, sag was different between ChCs and BCs in both species (Figure 2). Hyperpolarizing sag is most likely produced by the I_h_ channels [36], [37]. Their presence in FS interneurons was previously demonstrated in the neocortex of rats [38] and monkeys [20]. I_h_ currents are also shown to contribute to neuronal excitability [36], [37]. In accordance with potentially higher expression of I_h_ channels in ChCs than in BCs, the former have more negative voltage threshold for action potential initiation in rat.

AP threshold also scored high for the “cell type” factor in ANOVA test (Table 1). Differences in voltage AP threshold can be produced by the differences in fast and slow K^+^ currents that can shunt Na^+^ currents and increase firing threshold [39], including Kv1 channels [40], [41]. In our previous publication, we showed that Kv1 channel blocker decreased AP threshold in rat FS BCs [20]. In addition, the observed ChCs-BCs differences in AP threshold can be defined by the inward Na^+^ and low-threshold T-type Ca^2+^ currents [39]. Differences in excitability between ChCs and BCs were also associated with lower rheobase values in ChCs than in BCs in monkey and for a combined population of cells (Table 1). A similar difference between ChCs and BCs in rheobase was demonstrated in mouse neocortex [10].

In this study, ChCs fired at a substantially higher frequency than BCs in both rat and monkey, similar to previous findings in ferret [15] and monkey PFC [18] where the same difference was shown for ChCs and linear arbor cells (that, presumably, largely correspond to FS BCs from this study). It is known that FS PV-positive interneurons are involved in the generation of gamma oscillations [42], [43]. A differential role of ChCs and BCs in generation of gamma oscillations which was demonstrated in rodent brain studies utilizing *in vivo* [44] and *in vitro* [45] approaches could potentially be explained by the differences in firing frequency between ChCs and BCs demonstrated in this study (Figure 3).

### ChCs and BCs differential role in the cortical circuitry

The unique role of ChCs in cortical circuitry stems from at least two important features of this cell type: 1) the subcellular location of their inputs to the axon initial segment of pyramidal cells, and 2) the evidence that their effects on pyramidal cells can be either hyperpolarizing or depolarizing depending on the level of circuit activity [9], [11]. Thus, while BCs control inputs of pyramidal cells, ChCs, with their presynaptic terminals strategically placed near the location where APs are initiated, more closely control output. In this study, we showed that ChCs and BCs have different firing patterns, including differences in firing frequency, first spike latency, and more pronounced adaptation of firing (Table 1). These differences in firing behavior could potentially be translated into differential contribution of ChCs and BCs to rhythmic cortical activity. Indeed, Klausberger et al. demonstrated that in rat prefrontal cortex *in vivo* ChCs but not BCs regulate pyramidal cell activity in response to incoming excitation during transition from slow to theta oscillations [44]. Alternatively, BCs demonstrated more intense firing during the gamma-frequency dominated phases of the UP-states, which indicates their notable role in control of prefrontal gamma oscillations [44]. Another study performed in vitro showed that ChCs but not BCs maintain functional polarization of pyramidal cells during high-frequency gamma oscillations through separation of axonal and somatodendritic compartments that discharge at high-and low-frequency respectively [45]. It was suggested that high frequency firing of ChCs is necessary to provide tonic inhibition of pyramidal cell axon initial segment that maintains this separation [45].

In conclusion, the differences in electrophysiological membrane properties between the two types of FS interneurons, ChCs and BCs, from monkey and rat PFC described here can contribute to their differential behavior in normal cortical circuitry, e.g. generation of rhythmic brain activity [44], [45], as well as to their differential role in diseased brain. Thus, comparisons of postmortem brains from schizophrenia and control subjects indicate different disease-related alterations in ChCs and BCs; pre- and postsynaptic changes in ChCs-pyramidal synapses suggest their increased efficacy, whereas pre- and postsynaptic changes in BCs-pyramidal synapses suggest decreased input from BCs [46].

## References

[1] Ascoli GA, Alonso-Nanclares L, Anderson SA, Barrionuevo G, Benavides-Piccione R, et al (2008) Petilla terminology: nomenclature of features of GABAergic interneurons of the cerebral cortex. Nat Rev Neurosci 9: 557–568. 18568015 10.1038/nrn2402 PMC2868386

[2] Simons DJ (1978) Response properties of vibrissa units in rat SI somatosensory neocortex. J Neurophysiol 41: 798–820. 660231 10.1152/jn.1978.41.3.798

[3] McCormick DA, Connors BW, Lighthall JW, Prince DA (1985) Comparative electrophysiology of pyramidal and sparsely spiny stellate neurons of the neocortex. J Neurophysiol 54: 782–806. 2999347 10.1152/jn.1985.54.4.782

[4] Kawaguchi Y (1995) Physiological subgroups of nonpyramidal cells with specific morphological characteristics in layer II/III of rat frontal cortex. J Neurosci 15: 2638–2655. 7722619 10.1523/JNEUROSCI.15-04-02638.1995 PMC6577784

[5] Kawaguchi Y, Kubota Y (1997) GABAergic cell subtypes and their synaptic connections in rat frontal cortex. Cereb Cortex 7: 476–486. 9276173 10.1093/cercor/7.6.476

[6] Zaitsev AV, Gonzalez-Burgos G, Povysheva NV, Kroner S, Lewis DA, et al (2005) Localization of calcium-binding proteins in physiologically and morphologically characterized interneurons of monkey dorsolateral prefrontal cortex. Cereb Cortex 15: 1178–1186. 15590911 10.1093/cercor/bhh218

[7] Taniguchi H, Lu J, Huang ZJ (2012) The Spatial and Temporal Origin of Chandelier Cells in Mouse Neocortex. Science 10.1126/science.1227622 PMC4017638 23180771

[8] Inan M, Blazquez-Llorca L, Merchan-Perez A, Anderson SA, Defelipe J, et al (2013) Dense and overlapping innervation of pyramidal neurons by chandelier cells. J Neurosci 33: 1907–1914. 23365230 10.1523/JNEUROSCI.4049-12.2013 PMC3711719

[9] Szabadics J, Varga C, Molnar G, Olah S, Barzo P, et al (2006) Excitatory effect of GABAergic axo-axonic cells in cortical microcircuits. Science 311: 233–235. 16410524 10.1126/science.1121325

[10] Woodruff A, Xu Q, Anderson SA, Yuste R (2009) Depolarizing effect of neocortical chandelier neurons. Front Neural Circuits 3: 15. 19876404 10.3389/neuro.04.015.2009 PMC2769545

[11] Woodruff AR, McGarry LM, Vogels TP, Inan M, Anderson SA, et al (2011) State-dependent function of neocortical chandelier cells. J Neurosci 31: 17872–17886. 22159102 10.1523/JNEUROSCI.3894-11.2011 PMC4071969

[12] Glickfeld LL, Roberts JD, Somogyi P, Scanziani M (2009) Interneurons hyperpolarize pyramidal cells along their entire somatodendritic axis. Nat Neurosci 12: 21–23. 19029887 10.1038/nn.2230 PMC3505023

[13] Sauer JF, Struber M, Bartos M (2012) Interneurons provide circuit-specific depolarization and hyperpolarization. J Neurosci 32: 4224–4229. 22442084 10.1523/JNEUROSCI.5702-11.2012 PMC6621207

[14] Xu X, Callaway EM (2009) Laminar specificity of functional input to distinct types of inhibitory cortical neurons. J Neurosci 29: 70–85. 19129386 10.1523/JNEUROSCI.4104-08.2009 PMC2656387

[15] Krimer LS, Goldman-Rakic PS (2001) Prefrontal microcircuits: membrane properties and excitatory input of local, medium, and wide arbor interneurons. J Neurosci 21: 3788–3796. 11356867 10.1523/JNEUROSCI.21-11-03788.2001 PMC6762691

[16] Gonzalez-Burgos G, Krimer LS, Povysheva NV, Barrionuevo G, Lewis DA (2005) Functional properties of fast spiking interneurons and their synaptic connections with pyramidal cells in primate dorsolateral prefrontal cortex. J Neurophysiol 93: 942–953. 15385591 10.1152/jn.00787.2004

[17] Krimer LS, Zaitsev AV, Czanner G, Kroner S, Gonzalez-Burgos G, et al (2005) Cluster analysis-based physiological classification and morphological properties of inhibitory neurons in layers 2–3 of monkey dorsolateral prefrontal cortex. J Neurophysiol 94: 3009–3022. 15987765 10.1152/jn.00156.2005

[18] Zaitsev AV, Povysheva NV, Gonzalez-Burgos G, Rotaru D, Fish KN, et al (2009) Interneuron diversity in layers 2–3 of monkey prefrontal cortex. Cereb Cortex 19: 1597–1615. 19015370 10.1093/cercor/bhn198 PMC2693619

[19] Seamans JK, Lapish CC, Durstewitz D (2008) Comparing the prefrontal cortex of rats and primates: insights from electrophysiology. Neurotox Res 14: 249–262. 19073430 10.1007/BF03033814

[20] Povysheva NV, Zaitsev AV, Rotaru DC, Gonzalez-Burgos G, Lewis DA, et al (2008) Parvalbumin-positive basket interneurons in monkey and rat prefrontal cortex. J Neurophysiol 100: 2348–2360. 18632882 10.1152/jn.90396.2008 PMC2576192

[21] Thomson AM, West DC, Hahn J, Deuchars J (1996) Single axon IPSPs elicited in pyramidal cells by three classes of interneurones in slices of rat neocortex. J Physiol 496 Pt 1: 81–102. 8910198 10.1113/jphysiol.1996.sp021667 PMC1160826

[22] Lund JS, Lewis DA (1993) Local circuit neurons of developing and mature macaque prefrontal cortex: Golgi and immunocytochemical characteristics. J Comp Neurol 328: 282–312. 7678612 10.1002/cne.903280209

[23] Conde F, Lund JS, Jacobowitz DM, Baimbridge KG, Lewis DA (1994) Local circuit neurons immunoreactive for calretinin, calbindin D-28k or parvalbumin in monkey prefrontal cortex: distribution and morphology. J Comp Neurol 341: 95–116. 8006226 10.1002/cne.903410109

[24] Descalzo VF, Nowak LG, Brumberg JC, McCormick DA, Sanchez-Vives MV (2005) Slow adaptation in fast-spiking neurons of visual cortex. J Neurophysiol 93: 1111–1118. 15385594 10.1152/jn.00658.2004

[25] Breiman L (1984) Classification and regression trees. Belmont, Calif.: Wadsworth International Group. x, 358 p. p.

[26] Jung MW, Qin Y, McNaughton BL, Barnes CA (1998) Firing characteristics of deep layer neurons in prefrontal cortex in rats performing spatial working memory tasks. Cereb Cortex 8: 437–450. 9722087 10.1093/cercor/8.5.437

[27] Constantinidis C, Goldman-Rakic PS (2002) Correlated discharges among putative pyramidal neurons and interneurons in the primate prefrontal cortex. J Neurophysiol 88: 3487–3497. 12466463 10.1152/jn.00188.2002

[28] Wilson FA, O'Scalaidhe SP, Goldman-Rakic PS (1994) Functional synergism between putative gamma-aminobutyrate-containing neurons and pyramidal neurons in prefrontal cortex. Proc Natl Acad Sci U S A 91: 4009–4013. 8171027 10.1073/pnas.91.9.4009 PMC43712

[29] Steriade M, Timofeev I, Grenier F (2001) Natural waking and sleep states: a view from inside neocortical neurons. J Neurophysiol 85: 1969–1985. 11353014 10.1152/jn.2001.85.5.1969

[30] Douglas RJ, Martin KA (2004) Neuronal circuits of the neocortex. Annu Rev Neurosci 27: 419–451. 15217339 10.1146/annurev.neuro.27.070203.144152

[31] Gabbott PL, Dickie BG, Vaid RR, Headlam AJ, Bacon SJ (1997) Local-circuit neurones in the medial prefrontal cortex (areas 25, 32 and 24b) in the rat: morphology and quantitative distribution. J Comp Neurol 377: 465–499. 9007187 10.1002/(sici)1096-9861(19970127)377:4<465::aid-cne1>3.0.co;2-0

[32] Kawaguchi Y (1993) Physiological, morphological, and histochemical characterization of three classes of interneurons in rat neostriatum. J Neurosci 13: 4908–4923. 7693897 10.1523/JNEUROSCI.13-11-04908.1993 PMC6576359

[33] Sah P, Faber ES (2002) Channels underlying neuronal calcium-activated potassium currents. Prog Neurobiol 66: 345–353. 12015199 10.1016/s0301-0082(02)00004-7

[34] Sanchez-Vives MV, Nowak LG, McCormick DA (2000) Cellular mechanisms of long-lasting adaptation in visual cortical neurons in vitro. J Neurosci 20: 4286–4299. 10818164 10.1523/JNEUROSCI.20-11-04286.2000 PMC6772630

[35] Rudy B, Chow A, Lau D, Amarillo Y, Ozaita A, et al (1999) Contributions of Kv3 channels to neuronal excitability. Ann N Y Acad Sci 868: 304–343. 10414303 10.1111/j.1749-6632.1999.tb11295.x

[36] Pape HC (1996) Queer current and pacemaker: the hyperpolarization-activated cation current in neurons. Annu Rev Physiol 58: 299–327. 8815797 10.1146/annurev.ph.58.030196.001503

[37] Robinson RB, Siegelbaum SA (2003) Hyperpolarization-activated cation currents: from molecules to physiological function. Annu Rev Physiol 65: 453–480. 12471170 10.1146/annurev.physiol.65.092101.142734

[38] Aponte Y, Lien CC, Reisinger E, Jonas P (2006) Hyperpolarization-activated cation channels in fast-spiking interneurons of rat hippocampus. J Physiol 574: 229–243. 16690716 10.1113/jphysiol.2005.104042 PMC1817792

[39] Yang CR, Seamans JK, Gorelova N (1996) Electrophysiological and morphological properties of layers V–VI principal pyramidal cells in rat prefrontal cortex in vitro. J Neurosci 16: 1904–1921. 8774458 10.1523/JNEUROSCI.16-05-01904.1996 PMC6578693

[40] Glazebrook PA, Ramirez AN, Schild JH, Shieh CC, Doan T, et al (2002) Potassium channels Kv1.1, Kv1.2 and Kv1.6 influence excitability of rat visceral sensory neurons. J Physiol 541: 467–482. 12042352 10.1113/jphysiol.2001.018333 PMC2290329

[41] Guan D, Lee JC, Tkatch T, Surmeier DJ, Armstrong WE, et al (2006) Expression and biophysical properties of Kv1 channels in supragranular neocortical pyramidal neurones. J Physiol 571: 371–389. 16373387 10.1113/jphysiol.2005.097006 PMC1796796

[42] Bartos M, Vida I, Jonas P (2007) Synaptic mechanisms of synchronized gamma oscillations in inhibitory interneuron networks. Nat Rev Neurosci 8: 45–56. 17180162 10.1038/nrn2044

[43] Sohal VS, Zhang F, Yizhar O, Deisseroth K (2009) Parvalbumin neurons and gamma rhythms enhance cortical circuit performance. Nature 459: 698–702. 19396159 10.1038/nature07991 PMC3969859

[44] Massi L, Lagler M, Hartwich K, Borhegyi Z, Somogyi P, et al (2012) Temporal dynamics of parvalbumin-expressing axo-axonic and basket cells in the rat medial prefrontal cortex in vivo. J Neurosci 32: 16496–16502. 23152631 10.1523/JNEUROSCI.3475-12.2012 PMC4487822

[45] Dugladze T, Schmitz D, Whittington MA, Vida I, Gloveli T (2012) Segregation of axonal and somatic activity during fast network oscillations. Science 336: 1458–1461. 22700932 10.1126/science.1222017

[46] Lewis DA, Curley AA, Glausier JR, Volk DW (2012) Cortical parvalbumin interneurons and cognitive dysfunction in schizophrenia. Trends Neurosci 35: 57–67. 22154068 10.1016/j.tins.2011.10.004 PMC3253230

